# Socio-economic factors associated with mental health outcomes during the COVID-19 pandemic in South Korea

**DOI:** 10.3389/fpubh.2022.1024751

**Published:** 2022-12-13

**Authors:** Seo Yoon Lee, Jung Jae Lee, Hooyeon Lee

**Affiliations:** ^1^Department of Biobehavioral Nursing Science, College of Nursing, The University of Illinois at Chicago, Chicago, IL, United States; ^2^Department of Psychiatry, Dankook University Hospital, Cheonan, South Korea; ^3^Department of Psychiatry, College of Medicine, Dankook University, Cheonan, South Korea; ^4^Chungcheongnam-do Mental Health Welfare Center, Hongseong, South Korea; ^5^Department of Preventive Medicine, College of Medicine, The Catholic University of Korea, Seoul, South Korea

**Keywords:** COVID-19, pandemics, depression, anxiety, GAD-7 scale, PHQ-9, socio-ecological model of health, support system

## Abstract

**Background:**

Individuals are at an increased risk of adverse mental health outcomes during the COVID-19 pandemic. To reduce the impact on mental health outcomes that were induced by national-level policies, which may influence an individual at the community level, exploring the comprehensive relations between individual and environmental factors are needed. The aim is to examine socio-ecological factors associated with mental health outcomes, including depressive and anxiety symptoms, with the perspective of support to provide interventions that help the community during future disease outbreaks.

**Method:**

From 5 November to 20 November 2020, a cross-sectional and population-based study was conducted to assess the socio-ecological factors of mental health outcomes during the COVID-19 pandemic. A total of 1,000 participants, aged 20–69 years, in Chungnam Region, South Korea, were included in this study. Multiple linear regression models were used to examine the association between socio-ecological factors and mental health outcomes. The primary outcomes were individuals' mental health outcomes which are measured by PHQ-9 and GAD-7 scores.

**Results:**

Of the 1,000 participants, the average PHQ-9 was 4.39, and GAD-7 was 3.21 during the COVID-19 pandemic. Specifically, the participants with moderate or severe levels of PHQ-9 and GAD-7 were 12.6 and 6.8%, respectively. Higher levels of depressive and anxiety symptoms were associated with participants who were single, reported a lower household income, had decreased support from friends or family, and increased stress from the workplace or home. In subgroup analyses by age, gender, and household income, a similar trend was reported in individual and interpersonal-level factors. There were significant associations between regional-level factors, including gross regional domestic product (GRDP), mental health institutions, psychiatrists, nurse-to-population ratios, and individuals' mental health outcomes.

**Conclusion:**

The management of depressive and anxiety symptoms of individuals during the pandemic was better explained by individual and interpersonal characteristics rather than regional-level factors, highlighting the need for more policies aimed at these lower levels.

## Introduction

Due to the coronavirus disease 2019 (COVID-19) pandemic, countries have implemented various policies to prevent the spread, such as social distancing, multiple facility closures, and restrictions on travel. Such policies resulted in many changes in the lifestyles of people across the world. Before the COVID-19 pandemic, there was a case to be made for other infectious diseases (e.g., H1N1 and seasonal influenza) having an impact on public mental health (1, 2). There are multiple studies reporting the physical effects and sequelae, including myalgia, physical inactivity, cough, fever, breathing difficulty, and gastrointestinal symptoms caused by COVID-19 (3–6). Besides the physical effects, significant impacts on mental health including depression, anxiety, and suicidal ideation/behavior were reported around the world (7–17). In addition, unlike the physical effects, the impact on mental health not only affects individuals with direct virus contact but also the general public. In March 2020, in the US, the percentage of adults reporting the negative influence of COVID-19-related stress on their mental health was about 32% which increased to 53% by July 2020 (13). In South Korea, compared with the control group (i.e., matched individuals from the pre-pandemic period in 2019), individuals undergoing self-quarantine during the COVID-19 pandemic reported higher levels of depressive symptoms and a higher prevalence of major depression (14).

Among multiple methods to manage the population's mental health, enforcing a support system is one of the effective methods to implement for the public. There are multiple types of support defined (18). According to Cobb (19), although social support directly or indirectly affects mental health, depending on the type of support (i.e., instrumental, active, or material), the effect differs, and it is important to distinguish. Cobb's primary argument was that we should make a distinction between social support as perceived by an individual and social support as received in the form of resources or participation in supportive activities (18, 19). However, there was no study that investigated the effects of different types of support. Thus, in this study, individual- or interpersonal-level factors were focused on active support (i.e., marital status, support from friends and family, and stress from work and home) and regional-level factors were focused on instrumental or material support (i.e., mental health institution-to-population ratio and psychiatrist-to-population ratio).

Socio-ecological models were developed for a further understanding of the dynamic interrelations among various personal and environmental factors. Individual, interpersonal, and regional characteristics are likely to be connected to mental health outcomes among individuals in the community at various socio-ecological levels (20–22). To date, studies have provided evidence of the importance of factors at each level (22, 23). However, there is an existing gap confirming that, to our knowledge, no factors have been simultaneously examined at any socio-ecological level. Additional studies are needed to understand the range of socio-ecological factors contributing to mental health outcomes among community individuals in South Korea. Thus, this study aims to examine the association between socio-ecological factors (individual, interpersonal, and regional) and the individuals' mental health outcomes related to support in the general Korean population during the COVID-19 pandemic.

## Materials and methods

### Source of data and study population

This study used data from a cross-sectional survey targeting adults aged 20–69 years in the Chungnam Region of South Korea and investigated their mental health, experiences, and perceptions of the COVID-19 pandemic, and the psychosocial effects of COVID-19. Data were collected *via* conventional face-to-face interviews or an online survey panel. Embrain Public^®^ recruited the online survey panel through the random sampling of residential addresses throughout Korea at the end of October 2020. The participants answered the questionnaires anonymously *via* online survey or face-to-face interviews conducted from 5 November to 20 November 2020. Individuals voluntarily chose to participate by signing up on a panel platform. Online surveys or face-to-face interviews were conducted at the same time, and participants can be selected on how they would like to share their information. Of the 1,000 participants, 610 participated *via* online surveys, and 390 participated *via* face-to-face interviews. All participants provided informed consent prior to completing the study. The study included 16 Korean regional-level administrative districts, comprising seven cities (“Si”), seven counties (“Gun”), and two districts (“Gu”), to cover the Chungnam region.

### Dependent variable

The primary outcome variables were the participants' self-reported depression and anxiety levels. We used the Korean version of the Patient Health Questionnaire-9 (PHQ-9) to measure depressive status (24). The questionnaire consisted of nine items with a 4-point Likert scale ranging from 0 (never) to 3 (nearly every day) assessing the frequency of depressive symptoms over the past 2 weeks. The PHQ-9 score ranges from 0 to 27 points, and higher scores indicate more severe depression (25–27). The Korean version of the Generalized Anxiety Disorder-7 (GAD-7) scale was used to assess anxiety symptoms (28). The GAD-7 is composed of seven items assessing the frequency of anxiety over the past 2 weeks on a 4-point Likert scale ranging from 0 (never) to 3 (nearly every day). The total score of GAD-7 ranges from 0 to 21, and higher scores reflect greater severity of anxiety.

### Independent variable

#### Individual-level factors

Socioeconomic and interpersonal factors were selected based on previous studies on individual levels (15). Socioeconomic factors included age, gender, job status, marital status, average monthly household income, and education level. Since the survey was conducted using two different methods (i.e., face-to-face interview and online survey), the data collection method was controlled. As for interpersonal factors, the researchers collected the variables that measure the negative changes and the self-role disruptions in the participant's life due to COVID-19 (i.e., in social life and home). Specifically, the negative changes depend on whether the support from friends and family members has decreased and the stress from the workplace and home has increased.

#### Regional-level factors

Regional-level variables included the district's gross regional domestic product (GRDP), the community's mental health utilization indicators (i.e., mental health institution-to-population ratio, psychiatrist-to-population ratio, and nurse-to-population ratio), and the rates of unmet healthcare needs. GRDP is the sum of the market prices of all final goods and services produced within an administrative district for a certain period and is used to determine the economic structure or size. According to the European Parliament, an unmet healthcare need is defined as a state in which there is no existing satisfactory method of prevention, diagnosis, and treatment (29).

### Data analysis

**Figure 1:**
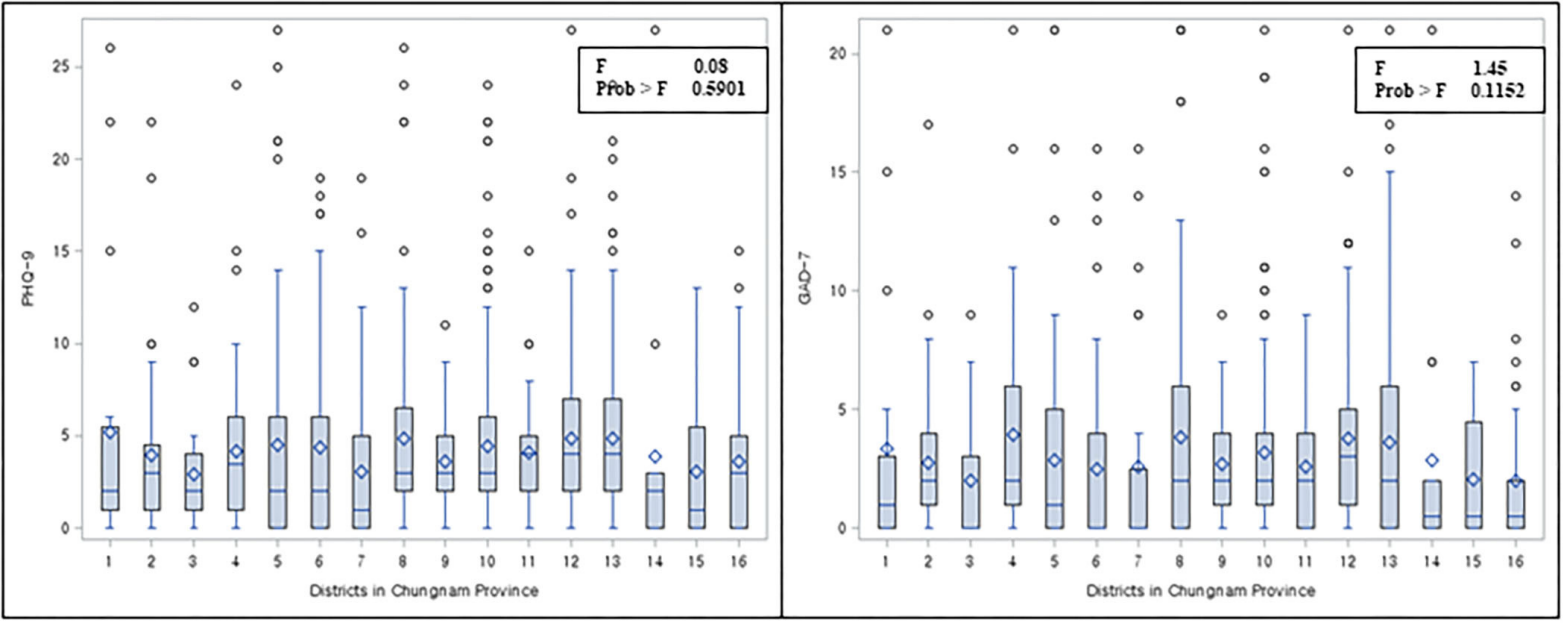
Distribution of PHQ-9 and GAD-7 by regional-level districts.

One-way ANOVA was conducted to determine whether there were any differences between the PHQ-9 and GAD-7 among districts (Figure 1). Although individuals were nested within region-level administrative districts of Chungnam Region, since there were no statistically significant differences between the districts, the generalized linear model was not employed. Descriptive statistics were conducted for all variables in the analysis (Table 1), and independent *t*-tests, one-way ANOVA, and chi-square tests were examined for differences between participants in the sample. Multicollinearity was tested, and the highest variance inflation factor (VIF) was 2.8, which is less than 4, meaning there was no excessive multicollinearity reported (30). We theorize that individual, interpersonal, and region-level factors predict changes in the individual's PHQ-9 and GAD-7. Each dependent variable (i.e., PHQ-9 and GAD-7) was modeled as a continuously distributed variable. Multiple linear regression was conducted to investigate the association between socio-ecological factors and mental health outcomes (Table 2). All socio-ecological factors were included simultaneously in adjusted models. Additionally, to investigate the differences in particular populations such as income, gender, and lifespan age, subgroup analyses were conducted (Tables 1–**5**, respectively). All statistical analyses were conducted in SAS version 9.4 (SAS Institute Inc., Cary, NC, USA). We considered a *p*-value of less than 0.05 to be statistically significant.

**Table 1:** Descriptive statistics of all variables in the analysis (*N* = 1,000).

**Characteristics**	***N***	**%**	**Mean**	**SD**
**Mental health status**				
PHQ-9	-	-	4.39	± 4.81
GAD-7	-	-	3.21	± 4.02
**Individual-level factors**				
Age (years)	-	-	45.03	± 13.41
**Gender**				
Male	528	(52.8)	-	-
Female	472	(47.2)	-	-
**Employment status**				
Unemployed	60	(6.0)	-	-
Employed	940	(94.0)	-	-
**Marital status**				
Single, separated, divorced, and widowed	327	(32.7)	-	-
Married, cohabiting and partnered	673	(67.3)	-	-
**Education**				
Lower than College graduate	394	(39.4)	-	-
College graduate or over	606	(60.6)	-	-
**Household income (USD per month)**				
< 2,000	104	(10.4)	-	-
2,000 ~ 4,999	603	(60.3)	-	-
≥ 5,000	293	(29.3)	-	-
**Survey method**				
Online	610	(61.0)	-	-
Interview	390	(39.0)	-	-
**Interpersonal-level factors**				
Severe interruption of their role in social life				
No	590	(59.0)	-	-
Yes	410	(41.0)	-	-
Severe interruption of their role at home				
No	872	(87.2)	-	-
Yes	128	(12.8)	-	-
Support from friends during COVID-19				
Same or increased	833	(83.3)	-	-
Decreased	167	(16.7)	-	-
Support from family during COVID-19				
Same or increased	923	(92.3)	-	-
Decreased	77	(7.7)	-	-
Stress from work during COVID-19				
Decreased or same	605	(60.5)	-	-
Increased	395	(39.5)	-	-
Stress from home during COVID-19				
Decreased or same	720	(72.0)	-	-
Increased	280	(28.0)	-	-
**Regional-level factors**				
Gross Regional Domestic Product (million Won)	-	-	15.80	± 10.46
Unmet healthcare need rates (%)	-	-	5.27	± 2.21
Mental health institution-to-population ratio^*^	-	-	3.83	± 1.57
Psychiatrist-to-population ratio^†^	-	-	5.95	± 2.87
Nurse-to-population ratio^*^	-	-	2.88	± 2.39

COVID-19, coronavirus disease 2019; GAD-7, Generalized Anxiety Disorder-7; PHQ-9, Patient Health Questionnaire-9; SD, Standard deviation;

* per 1,000 population;

† per 100,000 population.

**Table 2:** Multiple linear regression for the effect of individual and community factors on individual's mental health status.

	**PHQ-9**			**GAD-7**		
	**β**	**S.E**	***P*-value**	**β**	**S.E**	***P*-value**
**Individual-level factors**						
Intercept	**7.240**	0.691	< 0.0001	**5.594**	0.574	< 0.0001
Age (years)	−0.030	0.017	0.0803	−0.007	0.014	0.6437
**Gender**						
Male	Ref.	-	-	Ref.	-	-
Female	0.171	0.280	0.5414	0.447	0.233	0.0548
**Employment status**						
Unemployed	Ref.	-	-	Ref.	-	-
Employed	−1.147	0.613	0.0616	–**1.300**	0.509	0.0108
**Marital status**						
Single, separated, divorced, and widowed	Ref.	-	-	Ref.	-	-
Married, cohabiting and partnered	–**1.662**	0.405	< 0.0001	–**1.615**	0.337	< 0.0001
**Education**						
Lower than College graduate	Ref.	-	-	Ref.	-	-
College graduate or over	−0.575	0.307	0.0613	−0.423	0.255	0.0971
**Household income (USD per month)**						
< 2,000	Ref.	-	-	Ref.	-	-
2,000 ~ 4,999	–**1.361**	0.494	0.0060	–**0.910**	0.411	0.0270
≥ 5,000	–**1.616**	0.549	0.0033	–**1.102**	0.456	0.0159
**Survey method**						
Online	Ref.	-	-	Ref.	-	-
Interview	–**1.220**	0.399	0.0023	–**1.165**	0.332	0.0005
**Interpersonal-level factors**						
Severe interruption of their role in social life						
No	Ref.	-	-	Ref.	-	-
Yes	0.149	0.303	0.6223	0.311	0.252	0.2169
Severe interruption of their role at home						
No	Ref.	-	-	Ref.	-	-
Yes	0.243	0.452	0.5902	−0.184	0.376	0.6244
Support from friends during COVID-19						
Same or increased	Ref.	-	-	Ref.	-	-
Decreased	**1.286**	0.414	0.0019	**0.721**	0.344	0.0363
Support from family during COVID-19						
Same or increased	Ref.	-	-	Ref.	-	-
Decreased	**1.701**	0.574	0.0031	**1.549**	0.477	0.0012
Stress from work during COVID-19						
Decreased or same	Ref.	-	-	Ref.	-	-
Increased	**1.167**	0.299	< 0.0001	**0.972**	0.248	< 0.0001
Stress from home during COVID-19						
Decreased or same	Ref.	-	-	Ref.	-	-
Increased	**1.527**	0.334	< 0.0001	**1.715**	0.277	< 0.0001
**Regional-level factors**						
Gross Regional Domestic Product	0.010	0.016	0.5420	0.019	0.013	0.1536
Unmet healthcare need rates (%)	0.020	0.070	0.7702	−0.010	0.058	0.8579
Mental health institution-to-population ratio^*^	−0.028	0.133	0.8313	0.138	0.111	0.2135
Psychiatrist-to-population ratio^†^	−0.066	0.068	0.3335	−0.074	0.056	0.1893
Nurse-to-population ratio^*^	0.057	0.069	0.4134	0.041	0.057	0.4714

COVID-19, coronavirus disease 2019; GAD-7, Generalized Anxiety Disorder-7; PHQ-9, Patient Health Questionnaire-9; SD, Standard deviation;

* per 1,000 population;

† per 100,000 population.

Bold values indicates those are statistically significant.

## Results

The demographic characteristics of the study participants are provided in Table 1. The average age of the study participants was 45.03 years. Among 1,000 participants, 47.2% of them were women, 94% were employed, and 97% were college graduates. During COVID-19, 16.7% of the participants reported a decrease in support from friends, and 7.7% reported a decrease in family support. In addition, 39.5 and 28% of the participants reported that their stress from work and home increased, respectively. For regional-level variables, the average mental health institution-to-population ratio was 3.83 per 1,000 population, the psychiatrist-to-population ratio was 5.95 per 100,000 population, and the nurse-to-population ratio was 2.88 per 1,000 population. The mean of PHQ-9 and GAD-7 scores were 4.39 and 3.21, indicating a low level of depressive and anxiety symptoms, respectively. Each score distribution by each district is presented as a boxplot in Figure 1. No significant differences were identified between the districts (PHQ: *F* = 0.88, *p* = 0.5901; GAD-7: *F* = 1.45, *p* = 0.1152).

The result of the adjusted multiple linear regression analysis is presented in Table 2. The intercept of PHQ-9 and GAD-7 was 7.240 and 5.594, respectively. Among individual factors, those who were married, had an income over 2,000 USD, and who completed the survey face-to-face reported less depressive symptoms (PHQ-9) and anxiety (GAD-7). Participants who were employed showed fewer anxiety symptoms, but there was no statistically significant association reported with depressive symptoms. In addition, participants who reported a decrease in family and friend support and an increase in stress from work and home during COVID-19 reported higher depressive and anxiety symptoms. However, there was no statistically significant association shown between regional-level factors and both PHQ-9 and GAD-7 scores (Table 2).

Table 3 shows the subgroup analysis by lifespan age group. In the age group between 20 and 45, the association with PHQ-9, two individual factors, and three interpersonal factors were statistically significant, showing a similar trend. Two major factors, namely marital status and decreased support from friends, showed a bigger effect size. The association with GAD-7, two individual factors, and three interpersonal factors were statistically significant and showed a similar trend. Female participants reported more anxiety, which differed from the results of multiple linear regression. In the age group over 45, the association with PHQ-9, four individual factors, five interpersonal factors, and one regional-level factor were statistically significant. In the same age group, GAD-7 also showed similar significant associations except for four regional-level factors, including GRDP and healthcare utilization factors, which showed statistical significance. GRDP, the mental health institution-to-population ratio, and the nurse-to-population ratio showed a positive association, while the psychiatrists-to-population ratio showed a negative association.

**Table 3:** Subgroup analysis of multiple linear regression for the effect of individual and community factors on mental health status by lifespan age group.

	**PHQ-9**						**GAD-7**					
	**β**	**S.E**	***P*-value**	**β**	**S.E**	***P*-value**	**β**	**S.E**	***P*-value**	**β**	**S.E**	***P*-value**
	**20** ≤ **Age** ≤ **45**			**Age** > **45**			**20** ≤ **Age** ≤ **45**			**Age** > **45**		
**Individual-level factors**												
Intercept	**7.271**	1.019	< 0.0001	**7.849**	0.928	< 0.0001	**5.947**	0.854	< 0.0001	**5.744**	0.746	< 0.0001
**Gender**												
Male	Ref.	-	-	Ref.	-	-	Ref.	-	-	Ref.	-	-
Female	0.639	0.469	0.1732	−0.055	0.314	0.8598	**1.029**	0.393	0.0091	−0.101	0.253	0.6906
**Employment status**												
Unemployed	Ref.	-	-	Ref.	-	-	Ref.	-	-	Ref.	-	-
Employed	−0.649	0.918	0.4799	–**2.055**	0.781	0.0088	−1.260	0.769	0.1019	–**1.272**	0.628	0.0434
**Marital status**												
Single, separated, divorced, and widowed	Ref.	-	-	Ref.	-	-	Ref.	-	-	Ref.	-	-
Married, cohabiting and partnered	–**2.174**	0.492	< 0.0001	–**1.403**	0.589	0.0177	–**1.691**	0.413	< 0.0001	–**2.138**	0.474	< 0.0001
**Education**												
Lower than College graduate	Ref.	-	-	Ref.	-	-	Ref.	-	-	Ref.	-	-
College graduate or over	–**1.100**	0.535	0.0403	−0.071	0.334	0.8324	−0.863	0.448	0.0548	−0.242	0.269	0.3687
**Household income (USD per month)**												
< 2,000	Ref.	-	-	Ref.	-	-	Ref.	-	-	Ref.	-	-
2,000 ~ 4,999	−1.147	0.774	0.1389	–**1.671**	0.598	0.0054	−1.252	0.648	0.0540	−0.518	0.481	0.2816
≥ 5,000	−1.339	0.845	0.1138	–**1.861**	0.664	0.0053	−1.255	0.708	0.0770	−0.704	0.534	0.1885
**Survey method**												
Online	Ref.	-	-	Ref.	-	-	Ref.	-	-	Ref.	-	-
Interview	−0.709	1.035	0.4933	–**1.418**	0.364	0.0001	−1.641	0.867	0.0589	–**1.039**	0.293	0.0004
**Interpersonal-level factors**												
Severe interruption of their role in social life												
No	Ref.	-	-	Ref.	-	-	Ref.	-	-	Ref.	-	-
Yes	−0.474	0.490	0.3340	**0.896**	0.348	0.0104	−0.038	0.410	0.9255	**0.787**	0.280	0.0051
Severe interruption of their role at home												
No	Ref.	-	-	Ref.	-	-	Ref.	-	-	Ref.	-	-
Yes	0.348	0.696	0.6173	0.079	0.543	0.8840	−0.182	0.583	0.7545	−0.064	0.437	0.8831
Support from friends during COVID-19												
Same or increased	Ref.	-	-	Ref.	-	-	Ref.	-	-	Ref.	-	-
Decreased	**2.257**	0.647	0.0005	**0.000**	0.493	0.9999	**1.457**	0.543	0.0075	−0.360	0.396	0.3642
Support from family during COVID-19												
Same or increased	Ref.	-	-	Ref.	-	-	Ref.	-	-	Ref.	-	-
Decreased	1.261	0.876	0.1509	**1.647**	0.717	0.0220	1.037	0.734	0.1585	**1.845**	0.577	0.0015
Stress from work during COVID-19												
Decreased or same	Ref.	-	-	Ref.	-	-	Ref.	-	-	Ref.	-	-
Increased	**1.530**	0.485	0.0017	**0.795**	0.339	0.0196	**1.164**	0.406	0.0043	**0.810**	0.273	0.0032
Stress from home during COVID-19												
Decreased or same	Ref.	-	-	Ref.	-	-	Ref.	-	-	Ref.	-	-
Increased	**1.657**	0.541	0.0023	**1.298**	0.379	0.0007	**1.942**	0.453	< 0.0001	**1.361**	0.305	< 0.0001
**Regional-level factors**												
Gross Regional Domestic Product	−0.020	0.027	0.4658	**0.049**	0.018	0.0071	0.005	0.023	0.8250	**0.036**	0.015	0.0147
Unmet healthcare need rates (%)	−0.076	0.131	0.5584	0.105	0.071	0.1372	−0.128	0.109	0.2443	0.085	0.057	0.1372
Mental health institution-to-population ratio^*^	−0.316	0.240	0.1882	0.243	0.137	0.0775	0.040	0.201	0.8419	**0.242**	0.110	0.0285
Psychiatrist-to-population ratio^†^	−0.013	0.120	0.9153	−0.104	0.071	0.1425	−0.017	0.101	0.8684	–**0.137**	0.057	0.0165
Nurse-to-population ratio^*^	0.094	0.111	0.3967	0.033	0.081	0.6860	−0.072	0.093	0.4407	**0.192**	0.065	0.0033

COVID-19, coronavirus disease 2019; GAD-7, Generalized Anxiety Disorder-7; PHQ-9, Patient Health Questionnaire-9; SD, Standard deviation;

* per 1,000 population;

† per 100,000 population.

Bold values indicates those are statistically significant.

From Table 4, among the men, marital status, support from friends or family, and stress gained from home during COVID-19 were the main factors with a high impact on PHQ-9 and GAD-7. On the contrary, among the women, monthly household income and stress from home during COVID-19 showed significant associations with PHQ-9 and GAD-7. However, none of the regional-level factors showed a statistically significant association.

**Table 4:** Subgroup analysis of multiple linear regression for the effect of individual and community factors on mental health status by gender.

	**PHQ-9**						**GAD-7**					
	**β**	**S.E**	***P*-value**	**β**	**S.E**	***P*-value**	**β**	**S.E**	***P*-value**	**β**	**S.E**	***P*-value**
	**Male**			**Female**			**Male**			**Female**		
**Individual factors**												
Intercept	**6.523**	0.820	< 0.0001	**7.602**	1.478	< 0.0001	**4.883**	0.652	< 0.0001	**6.524**	1.279	< 0.0001
Age (years)	−0.011	0.022	0.6228	–**0.065**	0.028	0.0219	0.019	0.018	0.2898	–**0.051**	0.024	0.0391
**Employment status**												
Unemployed	Ref.	-	-	Ref.	-	-	Ref.	-	-	Ref.	-	-
Employed	–**1.557**	0.695	0.0256	−0.393	1.384	0.7764	–**1.615**	0.553	0.0036	−0.898	1.197	0.4537
**Marital status**												
Single, separated, divorced, and widowed	Ref.	-	-	Ref.	-	-	Ref.	-	-	Ref.	-	-
Married, cohabiting and partnered	–**1.974**	0.551	0.0004	−1.182	0.610	0.0534	–**1.700**	0.438	0.0001	–**1.438**	0.528	0.0067
**Education**												
Lower than College graduate	Ref.	-	-	Ref.	-	-	Ref.	-	-	Ref.	-	-
College graduate or over	−0.508	0.410	0.2154	−0.854	0.474	0.0720	−0.381	0.326	0.2429	−0.722	0.410	0.0788
**Household income (USD per month)**										
< 2,000	Ref.	-	-	Ref.	-	-	Ref.	-	-	Ref.	-	-
2,000 ~ 4,999	−0.424	0.718	0.5554	–**2.120**	0.696	0.0025	0.072	0.571	0.9002	–**1.706**	0.602	0.0048
≥ 5,000	−0.880	0.798	0.2706	–**2.236**	0.765	0.0037	−0.179	0.635	0.7775	–**1.846**	0.662	0.0055
**Survey method**												
Online	Ref.	-	-	Ref.	-	-	Ref.	-	-	Ref.	-	-
Interview	–**1.095**	0.496	0.0278	−1.167	0.689	0.0909	–**1.086**	0.394	0.0061	−0.956	0.596	0.1093
**Interpersonal factors**											
Severe interruption of their role in social life									
No	Ref.	-	-	Ref.	-	-	Ref.	-	-	Ref.	-	-
Yes	0.143	0.421	0.7338	−0.038	0.445	0.9328	0.163	0.335	0.6266	0.302	0.385	0.4336
Severe interruption of their role at home									
No	Ref.	-	-	Ref.	-	-	Ref.	-	-	Ref.	-	-
Yes	1.070	0.649	0.0999	−0.531	0.636	0.4041	0.283	0.516	0.5840	−0.513	0.550	0.3515
Support from friends during COVID-19										
Same or increased	Ref.	-	-	Ref.	-	-	Ref.	-	-	Ref.	-	-
Decreased	**1.238**	0.589	0.0361	**1.282**	0.592	0.0309	**0.979**	0.468	0.0371	0.457	0.512	0.3733
Support from family during COVID-19										
Same or increased	Ref.	-	-	Ref.	-	-	Ref.	-	-	Ref.	-	-
Decreased	**1.819**	0.809	0.0250	1.573	0.833	0.0596	**1.742**	0.643	0.0070	1.261	0.721	0.0808
Stress from work during COVID-19										
Decreased or same	Ref.	-	-	Ref.	-	-	Ref.	-	-	Ref.	-	-
Increased	**1.306**	0.402	0.0012	**1.039**	0.448	0.0210	**0.894**	0.319	0.0053	**0.989**	0.388	0.0111
Stress from home during COVID-19										
Decreased or same	Ref.	-	-	Ref.	-	-	Ref.	-	-	Ref.	-	-
Increased	**1.859**	0.476	0.0001	**1.304**	0.483	0.0072	**1.862**	0.378	< 0.0001	**1.594**	0.418	0.0002
**Regional-level factors**											
Gross Regional Domestic Product	0.027	0.022	0.2154	−0.005	0.025	0.8325	0.028	0.017	0.1088	0.012	0.021	0.5810
Unmet healthcare need rates (%)	0.066	0.093	0.4776	−0.016	0.105	0.8776	0.020	0.074	0.7828	−0.029	0.091	0.7497
Mental health institution-to-population ratio^*^	−0.250	0.180	0.1651	0.220	0.198	0.2686	0.013	0.143	0.9253	0.266	0.172	0.1216
Psychiatrist-to-population ratio^†^	0.016	0.092	0.8605	−0.151	0.101	0.1353	−0.072	0.073	0.3259	−0.074	0.087	0.3949
Nurse-to-population ratio^*^	0.002	0.093	0.9867	0.128	0.105	0.2232	0.012	0.074	0.8758	0.087	0.091	0.3403

COVID-19, coronavirus disease 2019; GAD-7, Generalized Anxiety Disorder-7; PHQ-9, Patient Health Questionnaire-9; SD, Standard deviation;

* per 1,000 population;

† per 100,000 population.

Bold values indicates those are statistically significant.

Finally, Table 5 shows subgroup analysis by monthly household incomes. Among the participants with incomes less than USD 2,000, decreased support from friends, and increased stress from home were significant factors of depressive symptoms (PHQ-9). On the other hand, individual characteristics such as marital and education statuses contributed to lowering anxiety levels (GAD-7). Finally, participants with more mental health resources in the region (i.e., the mental health institution-to-population ratio) reported fewer depressive symptoms and anxiety. The income of USD 2,000–4,999, employment status, survey method, decreased family support during COVID-19, and increased stress from work and home were statistically significant factors of PHQ-9 and GAD-7 among the groups. Factors particularly related to family or home were found to be significant among the participants with a household income of over USD 5,000.

**Table 5:** Subgroup analysis of multiple linear regression for the effect of individual and community factors on mental health status by monthly household income.

	**PHQ-9**									**GAD-7**								
	**β**	**S.E**	***P*-value**	**β**	**S.E**	***P*-value**	**β**	**S.E**	***P*-value**	**β**	**S.E**	***P*-value**	**β**	**S.E**	***P*-value**	**β**	**S.E**	***P*-value**
	<**USD 2,000**			**USD 2,000–4,999**			≥**USD 5,000**			<**USD 2,000**			**USD 2,000–4,999**			≥**USD 5,000**		
**Individual factors**																		
Intercept	**4.997**	1.709	0.0044	**6.495**	0.892	< 0.0001	3.564	1.918	0.0642	**4.371**	1.493	0.0044	**4.599**	0.738	< 0.0001	**3.675**	1.558	0.0190
Age (years)	−0.035	0.061	0.5657	–**0.048**	0.021	0.0249	0.017	0.033	0.6072	−0.013	0.053	0.8085	–**0.015**	0.018	0.4089	0.022	0.027	0.4276
**Gender**																		
Male	Ref.	-	-	Ref.	-	-	Ref.	-	-	Ref.	-	-	Ref.	-	-	Ref.	-	-
Female	1.895	1.352	0.1646	−0.039	0.339	0.9081	0.466	0.473	0.3249	2.307	1.181	0.0541	0.199	0.280	0.4789	0.555	0.384	0.1497
**Employment status**																		
Unemployed	Ref.	-	-	Ref.	-	-	Ref.	-	-	Ref.	-	-	Ref.	-	-	Ref.	-	-
Employed	−0.455	1.659	0.7844	–**2.367**	0.795	0.0030	2.309	1.691	0.1731	−0.780	1.450	0.5920	–**1.889**	0.658	0.0043	0.880	1.373	0.5223
**Marital status**																		
Single, separated, divorced, and widowed	Ref.	-	-	Ref.	-	-	Ref.	-	-	Ref.	-	-	Ref.	-	-	Ref.	-	-
Married, cohabiting and partnered	−3.335	1.754	0.0605	−0.646	0.471	0.1709	–**3.544**	0.772	< 0.0001	–**3.934**	1.532	0.0120	−0.732	0.390	0.0613	–**2.932**	0.627	< 0.0001
**Education**																		
Lower than College graduate	Ref.	-	-	Ref.	-	-	Ref.	-	-	Ref.	-	-	Ref.	-	-	Ref.	-	-
College graduate or over	−2.068	1.266	0.1061	–**0.775**	0.361	0.0320	0.345	0.600	0.5661	–**2.213**	1.107	0.0487	–**0.302**	0.299	0.3129	−0.190	0.488	0.6964
**Survey method**																		
Online	Ref.	-	-	Ref.	-	-	Ref.	-	-	Ref.	-	-	Ref.	-	-	Ref.	-	-
Interview	0.205	1.635	0.9005	–**1.592**	0.515	0.0021	−0.675	0.659	0.3068	−0.441	1.429	0.7586	–**1.373**	0.426	0.0014	−0.815	0.535	0.1289
**Interpersonal factors**																	
Severe interruption of their role in social life															
No	Ref.	-	-	Ref.	-	-	Ref.	-	-	Ref.	-	-	Ref.	-	-	Ref.	-	-
Yes	0.963	1.450	0.5085	0.269	0.366	0.4630	−0.049	0.516	0.9246	2.456	1.267	0.0560	0.272	0.303	0.3696	0.089	0.419	0.8323
Severe interruption of their role at home																
No	Ref.	-	-	Ref.	-	-	Ref.	-	-	Ref.	-	-	Ref.	-	-	Ref.	-	-
Yes	4.764	2.625	0.0731	0.882	0.531	0.0977	–**1.554**	0.778	0.0468	0.950	2.294	0.6798	0.407	0.440	0.3553	–**1.456**	0.632	0.0221
Support from friends during COVID-19																
Same or increased	Ref.	-	-	Ref.	-	-	Ref.	-	-	Ref.	-	-	Ref.	-	-	Ref.	-	-
Decreased	**3.379**	1.653	0.0440	0.849	0.488	0.0825	1.256	0.798	0.1166	2.740	1.445	0.0613	0.283	0.404	0.4844	0.782	0.648	0.2284
Support from family during COVID-19																
Same or increased	Ref.	-	-	Ref.	-	-	Ref.	-	-	Ref.	-	-	Ref.	-	-	Ref.	-	-
Decreased	2.019	2.370	0.3967	**1.509**	0.684	0.0279	**2.191**	1.111	0.0495	0.871	2.071	0.6752	**1.629**	0.567	0.0042	**2.105**	0.902	0.0204
Stress from work during COVID-19																
Decreased or same	Ref.	-	-	Ref.	-	-	Ref.	-	-	Ref.	-	-	Ref.	-	-	Ref.	-	-
Increased	1.525	1.533	0.3227	**1.755**	0.360	< 0.0001	0.124	0.496	0.8026	0.335	1.340	0.8035	**1.386**	0.298	< 0.0001	0.474	0.403	0.2401
Stress from home during COVID-19																
Decreased or same	Ref.	-	-	Ref.	-	-	Ref.	-	-	Ref.	-	-	Ref.	-	-	Ref.	-	-
Increased	**3.373**	1.571	0.0346	**1.002**	0.397	0.0119	**1.767**	0.593	0.0032	2.117	1.373	0.1267	**1.438**	0.329	< 0.0001	**1.899**	0.482	0.0001
**Regional-level factors**																	
Gross Regional Domestic Product	−0.146	0.078	0.0653	0.038	0.020	0.0523	0.012	0.027	0.6571	−0.123	0.068	0.0756	**0.051**	0.016	0.0020	0.000	0.022	0.9841
Unmet healthcare need rates (%)	−0.210	0.331	0.5280	0.081	0.079	0.3072	−0.079	0.142	0.5770	−0.243	0.289	0.4032	0.054	0.066	0.4094	−0.099	0.116	0.3928
Mental health institution-to-population ratio^*^	–**1.355**	0.593	0.0247	0.051	0.155	0.7406	0.396	0.252	0.1172	–**1.177**	0.518	0.0255	**0.260**	0.128	0.0427	0.357	0.205	0.0825
Psychiatrist-to-population ratio^†^	0.323	0.293	0.2734	−0.106	0.080	0.1832	−0.199	0.128	0.1211	0.349	0.256	0.1773	−0.124	0.066	0.0612	−0.144	0.104	0.1669
Nurse-to-population ratio^*^	0.428	0.380	0.2622	0.019	0.083	0.8231	0.017	0.116	0.8852	0.421	0.332	0.2077	−0.002	0.069	0.9809	0.031	0.094	0.7385

COVID-19, coronavirus disease 2019; GAD-7, Generalized Anxiety Disorder-7; PHQ-9, Patient Health Questionnaire-9; SD, Standard deviation;

* per 1,000 population;

† per 100,000 population;

N/A presents that the model did not converge.

Bold values indicates those are statistically significant.

## Discussion

This study aimed to examine the effects of individual, interpersonal, and regional factors at different socio-ecological levels on individuals' mental health status. The mean score of the depression level was 4.29, and the anxiety level was 3.29, indicating, in general, a very mild level of depression and anxiety (31). The average score of PHQ-9 in this study was reported higher compared to the reported results from previous studies (32), and the mean score of GAD-7 was also slightly higher compared to individuals with no mental disorder (28). With all the factors controlled, the intercept of predicted PHQ-9 and GAD-7 were mild-to-moderate levels of depression and anxiety. This difference might be attributable to the unique era of the pandemic and the situation of national-level lockdown and downsizing due to the COVID-19 case burden as compared to another era.

In addition to government measures, South Korea may have been able to effectively regulate COVID-19 because of public support. The current study found that factors at each level of the social-ecological model predicted the individual's depressive and anxiety symptoms during the pandemic in South Korea, confirming previous findings about the applicability of the social-ecological model to various issues (22, 33, 34). Our study results suggest that different efforts in multiple factors are needed to promote the management of mental health, in general. Moreover, although regional factors were identified as non-significant factors in general, for particular groups, such as groups by age and household income, regional factors, including GRDP and healthcare utilization variables, were revealed as significant factors. The factors significantly associated with anticipating the individual's mental health outcomes across the three levels were common in terms of support.

In this study, among intrapersonal factors, four of them were shown as significant factors in the associations with mental health outcomes throughout the analyses. Specifically, compared to the pre-pandemic era, the participants receiving less support from their family or friends or more stress from their workplace or home showed significant prediction with increased PHQ-9 and GAD-7 scores, which reflect a more severe level of depression and anxiety. Social bonds and supportive interactions with others are important for mental health; also, social support can buffer one from the negative consequences of stress (18). In addition, this study revealed the fact that among social support, family or home-related factors (i.e., marital status, support from family, and stress from home) have greater impacts on the individuals' depression and anxiety levels. These features were especially highlighted in several certain groups, including groups over 45 years old, or men, or with household incomes less than USD 2,000 or more than USD 5,000, whereas participants under 45 or with a household income less than USD 2,000 showed that support from friends was a significant factor.

An economic perspective can also be considered as one kind of social support, specifically material support. In general, participants with higher income levels showed lower levels of depressive and anxiety symptoms. Additionally, participants over 45 showed better depressive and anxiety levels in case of a secured financial status (i.e., employed or higher household income) (35–37). Among the women, household income status seemed to be the most significant predictor for their depressive and anxiety levels, whereas, among men, active support factors were significant. This finding may reflect cultural norms present in South Korea. In South Korea, wives are the main decision makers in the household and, therefore, may have been more affected by financial issues (38). In the subgroup analyses of the household incomes, each group highlighted different factors associated with the mental health outcomes of each group member. For example, the high-impact factors on mental health outcomes in low-income groups were related to financial support sources, whereas in middle-income groups, they were related to the security of the income sources (i.e., job status), and in high-income groups, they were associated with the family relationship. Based on the result interpretation of this study, active support has the main overall effect. However, the effect size may differ depending on the individual's income status since the material supports that affect the individuals' livelihood are different. Therefore, the support policy that considers people's SES is different, and its implementation is a matter to be considered.

Some subgroup analyses showed significant regional factors associated with mental health outcomes; however, those results were mixed. To understand the positive associations of regional factors with mental health status, we conducted an additional correlation analysis between socio-ecological factors and population as a proxy of urbanness and mental health outcomes (Supplementary Figure 1). The correlation result showed that population and mental health outcomes were associated with a higher GRDP and a higher ratio of healthcare personnel to the population. Among participants aged over 45, both PHQ-9 and GAD-7 showed positive associations with GRDP. GRDP measures the economic performance of a region which is an indicator of macro-economic performance in the local economy (39). Moreover, higher GRDP is most likely to be observed in metropolitan areas with large populations (39). In a previous study, participants living in metropolitan areas had lower perceived social support than those in rural areas (40). This is likely to be interpreted as urban communities tend to be more isolating or individualistic. The culture of familism may have decreased more in urban populations than in rural populations, as South Korea rapidly industrializes as a developing nation (40).

Among regional factors, healthcare utilization factors were the ones that showed mixed results; in other words, both positive and negative associations were shown. Specifically, those with a household income of less than 2,000 USD lived in areas with lower healthcare to an institution-to-population ratio and reported higher scores on the PHQ-9 and GAD-7. In South Korea, mental health promotion programs are planned and implemented in “Si,” “Gun,” and “Gu” to manage community mental health levels *via* community primary mental health clinics. Those programs are mandatory projects conducted in the clinics. According to our study, low household-income individuals are likely to benefit from resources such as greater numbers of mental health institutions provided in their community. Thus, a large supply of local mental health institutions can help provide mental illness prevention services and distribute and apply them prophylactically among low-income individuals (41). On the other hand, the psychiatrist-to-population ratio or nurse-to-population ratio was positively associated with mental health outcomes among participants aged over 45 and household income between 2,000 and 4,999. From the result of the correlation analysis, the psychiatrist-to-population ratio, nurse-to-population ratio, and household income showed positive correlations with the population. Considering those results, the beta coefficients showed in multiple regression analyses may reflect the characteristics of large populations.

This study is the first that examined the association of individual-, interpersonal-, and regional-level factors on the individuals' mental health outcomes in the general Korean population during the COVID-19 pandemic with the perspective of a socio-ecological model. There are some limitations that should be noted. First, this is a representative sample of one region, but not of the entire country or analyzing other regional differences, which threatens generalizability. Second, the main focus of this survey is on the individual and interpersonal factors impacting mental health status; however, the regional-level variables have not been developed with the purpose of the study in mind. Thus, we may omit key community factors with a significant impact on the individuals' depressive symptom management, including satisfaction with community mental health management programs. Third, the study measured mental symptoms using self-reported questionnaires rather than making clinical diagnoses. There may have also been response bias, with nervous individuals stressed by the epidemic and more inclined to engage in the survey. To minimize the response bias, the study survey was designed by a professional survey company with a psychiatrist consultant, and the interview was conducted by trained personnel, to avoid the leading questions. Fourth, since the survey's primary objective was to examine the general depressive mood and anxiety symptoms experienced by the community's residents as a result of COVID-19 during the pandemic, it is vital to evaluate for any pre-existing conditions that might have an impact on mental health or account for them in the analysis. However, the survey was more focused on environmental factors or general sociodemographic factors. The authors strongly suggest that in the next term of the survey, they should collect related pre-conditions that may influence mental health. Finally, due to the different survey methods, participants may have responded differently. There may be a possible social desirability bias (42). Social desirability bias is one of the response biases, the tendency of respondents to answer questions in a way that will be deemed positive by others. It is particularly observed in the questions that require socially desirable responses, which include personality, sexual behavior, and drug usage (42). To reduce the effect of bias, we confounded the survey method as a variable and conducted subgroup analysis by survey method to investigate different tendencies with beta coefficients that were not shown (Supplementary Table 1).

Despite these limitations, our findings have implications for psychological therapies aiming at lowering psychological distress and enhancing mental health and psychological resilience in the face of public health crises. To give evidence-based recommendations on responding to future pandemics in Korea, population-based research considering complex relations of the individual-, interpersonal-, and regional-level factors on mental health and COVID-19 should be established.

## Conclusion

In summary, we conducted a cross-sectional survey study to investigate socio-ecological factors associated with mental health outcomes, PHQ-9 and GAD-7, among 1,000 individuals during the COVID-19 pandemic in South Korea. From the adjusted multiple linear regression model, the study result revealed that the only different significant factor of PHQ-9 and GAD-7 was an individual-level factor which is employment status. Other than the employment status factor, three individual factors (i.e., marital status, monthly household income, and survey method) and four interpersonal factors (i.e., support from friends and family, stress from work and home) had significant associations, but no mental outcomes had significant associations with regional factors. All those factors are related to the economic status and support system of the individual and community. However, as shown in the previous study, individual and interpersonal factors were more adapted to explain the individuals' depressive and anxiety symptoms management during the COVID-19 pandemic than upper-level factors, suggesting the need for additional policies targeting these lower levels (22).

## Data availability statement

The raw data supporting the conclusions of this article will be made available by the authors, without undue reservation.

## Ethics statement

The studies involving human participants were reviewed and approved by the Institutional Review Board of Dankook University (DKU 2021-05-038). The patients/participants provided their written informed consent to participate in this study.

## Author contributions

SL has made substantial contributions to the conception, design of the work, interpretation of data, drafted the work, substantively revised it, final approval of the version to be published, and agreed to be accountable for the integrity of any part of the work are appropriately investigated and resolved. JL has made substantial contributions to the acquisition and interpretation of data for the work, revising it critically for important intellectual content, final approval of the version to be published, and agreed to be accountable for all aspects of the work in ensuring that questions related to the accuracy. HL has made substantial contributions to the design of the work, the acquisition, analysis, and interpretation of data, substantively revised the work, final approval of the version to be published, and agreed to be accountable for all aspects of the work in ensuring that questions related to the accuracy and integrity of any part of the work are appropriately investigated and resolved. All authors have approved the submitted version and have agreed to be personally accountable for the author's own contributions and to ensure that questions related to the accuracy or integrity of any part of the work, even ones in which the author was not personally involved, are appropriately investigated, resolved, and the resolution documented in the literature.
