# Supplementary material for: Socio-economic factors associated with mental health outcomes during the COVID-19 pandemic in South Korea
Source: Front Public Health. 2022 Dec 13;10:1024751. doi: 10.3389/fpubh.2022.1024751 (PMC9794092; doi:10.3389/fpubh.2022.1024751)
Supplement: Supplementary file 1 [file Table_1.DOCX]

**Supplemental Table**

| Supplemental Table 1. Subgroup analysis of multilevel linear regression for the effect of individual and community factors on mental health status by survey method | | | | | | | | | | | | | |
| --- | --- | --- | --- | --- | --- | --- | --- | --- | --- | --- | --- | --- | --- |
|  |  | PHQ-9 | | | | | | GAD-7 | | | | | |
|  |  | β | S.E | p-value | β | S.E | p-value | β | S.E | p-value | β | S.E | p-value |
|  |  | Online | | | Survey | | | Online | | | Survey | | |
| **Individual characteristics** | |  |  |  |  |  |  |  |  |  |  |  |  |
| Interncept | | **6.395** | 0.978 | <.0001 | **8.146** | 0.786 | <.0001 | **5.088** | 0.812 | <.0001 | **5.298** | 0.645 | <.0001 |
| Age (years) | | -0.021 | 0.026 | 0.4056 | -0.026 | 0.019 | 0.1813 | -0.017 | 0.021 | 0.4172 | **0.040** | 0.016 | 0.0121 |
| Gender | |  |  |  |  |  |  |  |  |  |  |  |  |
|  | Male | Ref. | - | - | Ref. | - | - | Ref. | - | - | Ref. | - | - |
|  | Female | 0.307 | 0.421 | 0.4653 | 0.419 | 0.298 | 0.1607 | 0.663 | 0.349 | 0.0582 | 0.143 | 0.245 | 0.5580 |
| Employment status | |  |  |  |  |  |  |  |  |  |  |  |  |
|  | Unemployed | Ref. | - | - | Ref. | - | - | Ref. | - | - | Ref. | - | - |
|  | Employed | 0.076 | 0.885 | 0.9314 | **-3.916** | 0.685 | <.0001 | -0.668 | 0.735 | 0.3631 | **-2.340** | 0.562 | <.0001 |
| Marital status | |  |  |  |  |  |  |  |  |  |  |  |  |
|  | Single, separated, divorced, and widowed | Ref. | - | - | Ref. | - | - | Ref. | - | - | Ref. | - | - |
|  | Married, cohabiting and partnered | **-1.840** | 0.534 | 0.0006 | **-1.644** | 0.619 | 0.0082 | **-1.469** | 0.443 | 0.0010 | **-2.905** | 0.508 | <.0001 |
| Education | |  |  |  |  |  |  |  |  |  |  |  |  |
|  | Lower than College graduate | Ref. | - | - | Ref. | - | - | Ref. | - | - | Ref. | - | - |
|  | College graduate or over | **-1.050** | 0.485 | 0.0308 | -0.012 | 0.312 | 0.9684 | **-0.793** | 0.403 | 0.0493 | 0.063 | 0.256 | 0.8072 |
| Household income (USD per month) | |  |  |  |  |  |  |  |  |  |  |  |  |
|  | < 2,000 | Ref. | - | - | Ref. | - | - | Ref. | - | - | Ref. | - | - |
|  | 2,000 ~ 4,999 | -1.266 | 0.723 | 0.0804 | **-1.197** | 0.564 | 0.0346 | -1.089 | 0.600 | 0.0699 | -0.084 | 0.463 | 0.8564 |
|  | ≥ 5,000 | **-1.735** | 0.786 | 0.0276 | -0.980 | 0.638 | 0.1254 | **-1.412** | 0.652 | 0.0308 | 0.196 | 0.524 | 0.7077 |
| **Interpersonal factors** | |  |  |  |  |  |  |  |  |  |  |  |  |
| Severe interruption of their role in social life | |  |  |  |  |  |  |  |  |  |  |  |  |
|  | No | Ref. | - | - | Ref. | - | - | Ref. | - | - | Ref. | - | - |
|  | Yes | -0.050 | 0.456 | 0.9132 | **0.677** | 0.315 | 0.0323 | 0.242 | 0.379 | 0.5223 | 0.489 | 0.258 | 0.0594 |
| Severe interruption of their role at home | |  |  |  |  |  |  |  |  |  |  |  |  |
|  | No | Ref. | - | - | Ref. | - | - | Ref. | - | - | Ref. | - | - |
|  | Yes | 0.697 | 0.624 | 0.2648 | -0.888 | 0.549 | 0.1067 | -0.124 | 0.518 | 0.8115 | -0.273 | 0.451 | 0.5450 |
| Support from friends during COVID-19 | |  |  |  |  |  |  |  |  |  |  |  |  |
|  | Same or increased | Ref. | - | - | Ref. | - | - | Ref. | - | - | Ref. | - | - |
|  | Decreased | **1.421** | 0.575 | 0.0137 | 0.878 | 0.491 | 0.0745 | 0.667 | 0.477 | 0.1625 | 0.723 | 0.403 | 0.0732 |
| Support from family during COVID-19 | |  |  |  |  |  |  |  |  |  |  |  |  |
|  | Same or increased | Ref. | - | - | Ref. | - | - | Ref. | - | - | Ref. | - | - |
|  | Decreased | 0.963 | 0.812 | 0.2360 | **2.900** | 0.659 | <.0001 | 0.922 | 0.674 | 0.1718 | **2.366** | 0.540 | <.0001 |
| Stress from work during COVID-19 | |  |  |  |  |  |  |  |  |  |  |  |  |
|  | Decreased or same | Ref. | - | - | Ref. | - | - | Ref. | - | - | Ref. | - | - |
|  | Increased | **1.466** | 0.442 | 0.0010 | **0.695** | 0.315 | 0.0280 | **1.268** | 0.367 | 0.0006 | **0.564** | 0.258 | 0.0296 |
| Stress from home during COVID-19 | |  |  |  |  |  |  |  |  |  |  |  |  |
|  | Decreased or same | Ref. | - | - | Ref. | - | - | Ref. | - | - | Ref. | - | - |
|  | Increased | **1.702** | 0.489 | 0.0005 | **0.904** | 0.362 | 0.0129 | **1.990** | 0.406 | <.0001 | **1.095** | 0.297 | 0.0003 |
| **Regional level factors** | |  |  |  |  |  |  |  |  |  |  |  |  |
| Gross Regional Domestic Product | | -0.007 | 0.024 | 0.7697 | **0.064** | 0.018 | 0.0003 | 0.015 | 0.020 | 0.4557 | **0.040** | 0.015 | 0.0069 |
| Unmet healthcare need rates (%) | | -0.076 | 0.117 | 0.5140 | 0.093 | 0.063 | 0.1394 | -0.105 | 0.097 | 0.2799 | 0.086 | 0.052 | 0.0956 |
| Mental health institution-to-population ratio* | | -0.191 | 0.208 | 0.3578 | **0.385** | 0.132 | 0.0039 | 0.076 | 0.172 | 0.6597 | **0.376** | 0.109 | 0.0006 |
| Psychiatrist-to-population ratio† | | -0.010 | 0.106 | 0.9238 | **-0.191** | 0.066 | 0.0038 | -0.038 | 0.088 | 0.6652 | **-0.166** | 0.054 | 0.0021 |
| Nurse-to-population ratio* | | 0.063 | 0.102 | 0.5370 | -0.021 | 0.075 | 0.7794 | -0.074 | 0.085 | 0.3821 | **0.187** | 0.062 | 0.0026 |
| *Note*: COVID-19, coronavirus disease 2019; GAD-7, Generalized Anxiety Disorder-7; PHQ-9, Patient Health Questionnaire-9; SD, Standard deviation; *: per 1,000 population; †: per 100,000 population | | | | | | | | | | | | | |

| **Supplemental Table 2. Descriptive statistics of all variables in the analysis by PHQ-9 level (N=1,000)** | | | | | | | | | | |
| --- | --- | --- | --- | --- | --- | --- | --- | --- | --- | --- |
| Characteristics | | None or minimal | | Mild | | Moderate | | Severe | | p-value |
|  |  | N / Mean | % / SD | N / Mean | % / SD | N / Mean | % / SD | N / Mean | % / SD |  |
| Total | | 643 | (64.3) | 231 | (23.1) | 80 | (8.0) | 46 | (4.6) | <.0001 |
| **Individual level factors** | |  |  |  |  |  |  |  |  |  |
| Age (years) | | 47.99 | ± 13.16 | 41.13 | ± 12.51 | 37.14 | ± 10.81 | 36.98 | ± 11.79 | <.0001 |
| Gender | |  |  |  |  |  |  |  |  |  |
|  | Male | 332 | (62.9) | 131 | (24.8) | 44 | (8.3) | 21 | (4.0) | 0.4126 |
|  | Female | 311 | (65.9) | 100 | (21.2) | 36 | (7.6) | 25 | (5.3) |  |
| Employment status | |  |  |  |  |  |  |  |  |  |
|  | Unemployed | 30 | (50.0) | 15 | (25.0) | 8 | (13.3) | 7 | (11.7) | 0.0101 |
|  | Employed | 613 | (65.2) | 216 | (23.0) | 72 | (7.7) | 39 | (4.2) |  |
| Marital status | |  |  |  |  |  |  |  |  |  |
|  | Single, separated, divorced, and widowed | 147 | (45.0) | 102 | (31.2) | 48 | (14.7) | 30 | (9.2) | <.0001 |
|  | Married, cohabiting and partnered | 496 | (73.7) | 129 | (19.2) | 32 | (4.8) | 16 | (2.4) |  |
| Education | |  |  |  |  |  |  |  |  |  |
|  | Lower than College graduate | 264 | (67.0) | 82 | (20.8) | 27 | (6.9) | 21 | (5.3) | 0.2576 |
|  | College graduate or over | 379 | (62.5) | 149 | (24.6) | 53 | (8.8) | 25 | (4.1) |  |
| Household income (USD per month) | |  |  |  |  |  |  |  |  |  |
|  | < 2,000 | 53 | (51.0) | 26 | (25.0) | 11 | (10.6) | 14 | (13.5) | 0.0001 |
|  | 2,000 ~ 4,999 | 388 | (64.3) | 142 | (23.6) | 52 | (8.6) | 21 | (3.5) |  |
|  | ≥ 5,000 | 202 | (68.9) | 63 | (21.5) | 17 | (5.8) | 11 | (3.8) |  |
| Survey method | |  |  |  |  |  |  |  |  |  |
|  | Online | 332 | (54.4) | 168 | (27.5) | 68 | (11.2) | 42 | (6.9) | <.0001 |
|  | Interview | 311 | (79.7) | 63 | (16.2) | 12 | (3.1) | 4 | (1.0) |  |
| **Interpersonal factors** | |  |  |  |  |  |  |  |  |  |
| Severe interruption of their role in social life | |  |  |  |  |  |  |  |  |  |
|  | No | 408 | (69.2) | 118 | (20.0) | 40 | (6.8) | 24 | (4.1) | 0.0020 |
|  | Yes | 235 | (57.3) | 113 | (27.6) | 40 | (9.8) | 22 | (5.4) |  |
| Severe interruption of their role at home | |  |  |  |  |  |  |  |  |  |
|  | No | 572 | (65.6) | 197 | (22.6) | 68 | (7.8) | 35 | (4.0) | 0.0464 |
|  | Yes | 71 | (55.5) | 34 | (26.6) | 12 | (9.4) | 11 | (8.6) |  |
| Support from friends during COVID-19 | |  |  |  |  |  |  |  |  |  |
|  | Same or increased | 577 | (69.3) | 169 | (20.3) | 55 | (6.6) | 32 | (3.8) | <.0001 |
|  | Decreased | 66 | (39.5) | 62 | (37.1) | 25 | (15.0) | 14 | (8.4) |  |
| Support from family during COVID-19 | |  |  |  |  |  |  |  |  |  |
|  | Same or increased | 615 | (66.6) | 207 | (22.4) | 64 | (6.9) | 37 | (4.0) | <.0001 |
|  | Decreased | 28 | (36.4) | 24 | (31.2) | 16 | (20.8) | 9 | (11.7) |  |
| Stress from work during COVID-19 | |  |  |  |  |  |  |  |  |  |
|  | Decreased or same | 416 | (68.8) | 126 | (20.8) | 37 | (6.1) | 26 | (4.3) | 0.0016 |
|  | Increased | 227 | (57.5) | 105 | (26.6) | 43 | (10.9) | 20 | (5.1) |  |
| Stress from home during COVID-19 | |  |  |  |  |  |  |  |  |  |
|  | Decreased or same | 498 | (69.2) | 158 | (21.9) | 39 | (5.4) | 25 | (3.5) | <.0001 |
|  | Increased | 145 | (51.8) | 73 | (26.1) | 41 | (14.6) | 21 | (7.5) |  |
| **Regional level factors** | |  |  |  |  |  |  |  |  |  |
| Gross Regional Domestic Product (million Won) | | 15.39 | ± 10.66 | 16.29 | ± 10.06 | 17.94 | ± 9.73 | 15.28 | ± 10.68 | 0.1745 |
| Unmet healthcare need rates (%) | | 5.27 | ± 2.22 | 5.29 | ± 2.32 | 5.22 | ± 2.06 | 5.32 | ± 1.83 | 0.9944 |
| Mental health institution-to-population ratio* | | 3.84 | ± 1.58 | 3.88 | ± 1.57 | 3.70 | ± 1.45 | 3.63 | ± 1.56 | 0.6696 |
| Psychiatrist-to-population ratio† | | 5.99 | ± 2.95 | 5.90 | ± 2.73 | 5.97 | ± 2.42 | 5.62 | ± 3.18 | 0.8406 |
| Nurse-to-population ratio* | | 2.79 | ± 2.29 | 3.12 | ± 2.62 | 3.20 | ± 2.66 | 2.30 | ± 1.91 | 0.0601 |
| *Note*: COVID-19, coronavirus disease 2019; GAD-7, Generalized Anxiety Disorder-7; PHQ-9, Patient Health Questionnaire-9; SD, Standard deviation; *: per 1,000 population; †: per 100,000 population | | | | | | | | | | |

| **Supplemental Table 3. Descriptive statistics of all variables in the analysis by GAD-7 level (N=1,000)** | | | | | | | | | | |
| --- | --- | --- | --- | --- | --- | --- | --- | --- | --- | --- |
| Characteristics | | Mild | | Mild | | Moderate | | Severe | | p-value |
|  |  | N / Mean | % / SD | N / Mean | % / SD | N / Mean | % / SD | N / Mean | % / SD |  |
| Total | | 734 | (73.4) | 198 | (19.8) | 39 | (3.9) | 29 | (2.9) | <.0001 |
| **Individual level factors** | |  |  |  |  |  |  |  |  |  |
| Age (years) | | 47.19 | ± 13.12 | 39.75 | ± 12.57 | 37.28 | ± 12.82 | 37.00 | ± 10.51 | <.0001 |
| Gender | |  |  |  |  |  |  |  |  |  |
|  | Male | 338 | (75.0) | 95 | (19.5) | 18 | (3.4) | 11 | (2.1) | 0.2954 |
|  | Female | 396 | (71.6) | 103 | (20.1) | 21 | (3.9) | 18 | (2.9) |  |
| Employment status | |  |  |  |  |  |  |  |  |  |
|  | Unemployed | 36 | (60.0) | 12 | (20.0) | 6 | (10.0) | 6 | (10.0) | 0.0003 |
|  | Employed | 698 | (74.3) | 186 | (19.8) | 33 | (3.5) | 33 | (2.5) |  |
| Marital status | |  |  |  |  |  |  |  |  |  |
|  | Single, separated, divorced, and widowed | 179 | (54.7) | 102 | (31.2) | 25 | (7.7) | 21 | (6.4) | <.0001 |
|  | Married, cohabiting and partnered | 555 | (82.5) | 96 | (14.3) | 14 | (2.1) | 8 | (1.2) |  |
| Education | |  |  |  |  |  |  |  |  |  |
|  | Lower than College graduate | 295 | (74.9) | 71 | (18.0) | 17 | (4.3) | 11 | (2.8) | 0.6719 |
|  | College graduate or over | 439 | (72.4) | 127 | (21.0) | 22 | (3.6) | 18 | (3.0) |  |
| Household income (USD per month) | |  |  |  |  |  |  |  |  |  |
|  | < 2,000 | 65 | (62.5) | 21 | (20.2) | 8 | (7.7) | 10 | (9.6) | 0.0002 |
|  | 2,000 ~ 4,999 | 443 | (73.5) | 126 | (20.9) | 21 | (3.5) | 13 | (2.2) |  |
|  | ≥ 5,000 | 226 | (77.1) | 51 | (17.4) | 10 | (3.4) | 6 | (2.1) |  |
| Survey method | |  |  |  |  |  |  |  |  |  |
|  | Online | 395 | (64.8) | 155 | (25.4) | 34 | (5.6) | 26 | (4.3) | <.0001 |
|  | Interview | 339 | (86.9) | 43 | (11.0) | 5 | (1.3) | 3 | (0.8) |  |
| **Interpersonal factors** | |  |  |  |  |  |  |  |  |  |
| Severe interruption of their role in social life | |  |  |  |  |  |  |  |  |  |
|  | No | 454 | (77.0) | 102 | (17.3) | 21 | (3.6) | 13 | (2.2) | 0.0195 |
|  | Yes | 280 | (68.3) | 96 | (23.4) | 18 | (4.4) | 16 | (3.9) |  |
| Severe interruption of their role at home | |  |  |  |  |  |  |  |  |  |
|  | No | 654 | (75.0) | 162 | (18.6) | 31 | (3.6) | 25 | (2.9) | 0.0221 |
|  | Yes | 80 | (62.5) | 36 | (28.1) | 8 | (6.3) | 4 | (3.1) |  |
| Support from friends during COVID-19 | |  |  |  |  |  |  |  |  |  |
|  | Same or increased | 644 | (77.3) | 141 | (16.9) | 28 | (3.4) | 20 | (2.4) | <.0001 |
|  | Decreased | 90 | (53.9) | 57 | (34.1) | 11 | (6.6) | 9 | (5.4) |  |
| Support from family during COVID-19 | |  |  |  |  |  |  |  |  |  |
|  | Same or increased | 697 | (75.5) | 173 | (18.7) | 30 | (3.3) | 23 | (2.5) | <.0001 |
|  | Decreased | 37 | (48.1) | 25 | (32.5) | 9 | (11.7) | 6 | (7.8) |  |
| Stress from work during COVID-19 | |  |  |  |  |  |  |  |  |  |
|  | Decreased or same | 477 | (78.8) | 93 | (15.4) | 18 | (3.0) | 17 | (2.8) | <.0001 |
|  | Increased | 257 | (65.1) | 105 | (26.6) | 21 | (5.3) | 12 | (3.0) |  |
| Stress from home during COVID-19 | |  |  |  |  |  |  |  |  |  |
|  | Decreased or same | 571 | (79.3) | 117 | (16.3) | 18 | (2.5) | 14 | (1.9) | <.0001 |
|  | Increased | 163 | (58.2) | 81 | (28.9) | 21 | (7.5) | 15 | (5.4) |  |
| **Regional level factors** | |  |  |  |  |  |  |  |  |  |
| Gross Regional Domestic Product (million Won) | | 15.31 | ± 10.58 | 17.06 | ± 10.00 | 17.75 | ± 10.16 | 16.89 | ± 10.26 | 0.1039 |
| Unmet healthcare need rates (%) | | 5.32 | ± 2.26 | 5.22 | ± 2.28 | 4.93 | ± 1.35 | 4.88 | ± 1.32 | 0.5208 |
| Mental health institution-to-population ratio* | | 3.83 | ± 1.58 | 3.81 | ± 1.55 | 4.08 | ± 1.43 | 3.61 | ± 1.62 | 0.6576 |
| Psychiatrist-to-population ratio† | | 5.97 | ± 2.94 | 5.83 | ± 2.62 | 6.64 | ± 2.40 | 5.41 | ± 3.20 | 0.3064 |
| Nurse-to-population ratio* | | 2.81 | ± 2.32 | 3.10 | ± 2.60 | 3.45 | ± 2.79 | 2.34 | ± 1.94 | 0.1104 |
| *Note*: COVID-19, coronavirus disease 2019; GAD-7, Generalized Anxiety Disorder-7; PHQ-9, Patient Health Questionnaire-9; SD, Standard deviation; *: per 1,000 population; †: per 100,000 population | | | | | | | | | | |

**Figure legend**

Supplemental Figure 1. Correlation coefficients of components to PHQ-9, GAD-7, and population at the individual and regional level factors
