# Supplementary figures and images for: Socio-economic factors associated with mental health outcomes during the COVID-19 pandemic in South Korea
Source: Front Public Health. 2022 Dec 13;10:1024751. doi: 10.3389/fpubh.2022.1024751 (PMC9794092; doi:10.3389/fpubh.2022.1024751)

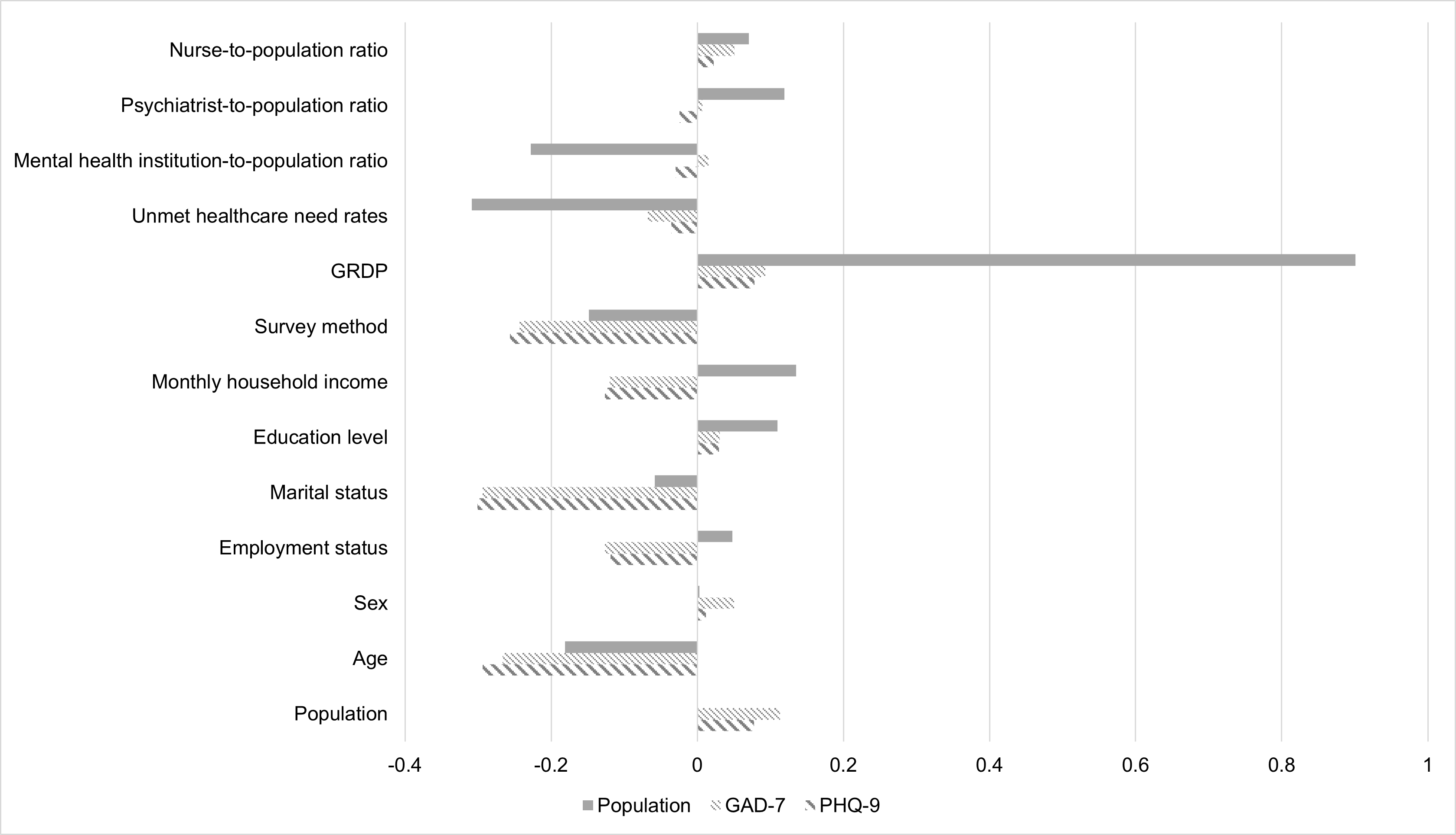

Supplement: Supplementary file 2 [file Image_1.TIF]
